# Tetra­gonal polymorph of 5,5-dichloro­barbituric acid

**DOI:** 10.1107/S1600536811054626

**Published:** 2011-12-23

**Authors:** Thomas Gelbrich, Denise Rossi, Ulrich J. Griesser

**Affiliations:** a Institute of Pharmacy, University of Innsbruck, Innrain 52c, 6020 Innsbruck, Austria

## Abstract

The tetra­gonal polymorph of 5,5-dichloro­barbituric acid (m.p. 478 K), C_4_H_2_Cl_2_N_2_O_3_, forms an N—H⋯O hydrogen-bonded tape structure along [001]. Two tapes related by a twofold rotation axis are associated *via* Cl⋯O contacts [3.201 (1) Å], and four such chain pairs are arranged around a fourfold roto-inversion axis. The crystal structures of the monoclinic and ortho­rhom­bic polymorphs have been reported previously [Gelbrich *et al.* (2011 ▶). *CrystEngComm*, **13**, 5502–5509].

## Related literature

The polymorphic nature of 5,5-dichloro­barbituric acid was mentioned in Groth’s compendium on the chemical crystallography of organic compounds, published more than a hundred years ago (Groth, 1910 ▶). For the monoclinic and ortho­rhom­bic polymorphs, see: Gelbrich *et al.* (2011 ▶). For related structures, see: Gartland & Craven (1971 ▶); Gelbrich *et al.* (2007 ▶, 2010 ▶, 2010*a* ▶,*b* ▶); Nichol & Clegg (2007 ▶); Zencirci *et al.* (2009 ▶, 2010 ▶); DesMarteau *et al.* (1994 ▶). For a description of the synthesis, see: Ziegler *et al.* (1962 ▶). For hydrogen-bond motifs, see: Bernstein *et al.* (1995 ▶); Etter *et al.* (1990 ▶).
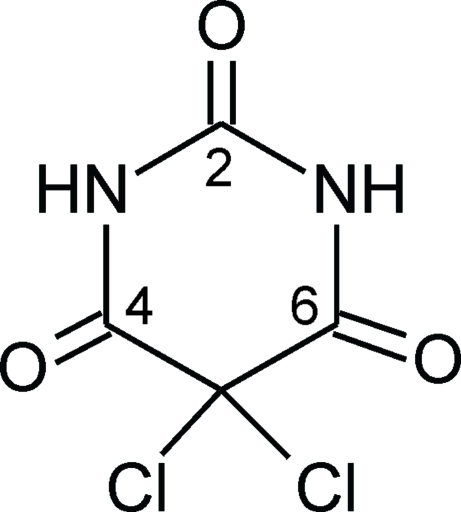


## Experimental

### 

#### Crystal data

C_4_H_2_Cl_2_N_2_O_3_
*M* *_r_* = 196.98
Tetragonal, 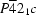

*a* = 13.8883 (3) Å
*c* = 6.9126 (2) Å
*V* = 1333.34 (6) Å^3^
*Z* = 8
Mo *K*α radiation
μ = 0.92 mm^−1^
*T* = 173 K
0.20 × 0.05 × 0.05 mm

#### Data collection

Oxford Diffraction Xcalibur Ruby Gemini ultra diffractometer
Absorption correction: multi-scan (*CrysAlis RED*; Oxford Diffraction, 2003 ▶) *T*_min_ = 0.837, *T*_max_ = 0.955
11025 measured reflections
1310 independent reflections
1242 reflections with *I* > 2σ(*I*)
*R*_int_ = 0.041

#### Refinement

*R*[*F* ^2^ > 2σ(*F* ^2^)] = 0.020
*wR*(*F* ^2^) = 0.050
*S* = 1.07
1310 reflections
107 parameters
2 restraints
All H-atom parameters refined
Δρ_max_ = 0.20 e Å^−3^
Δρ_min_ = −0.16 e Å^−3^
Absolute structure: Flack (1983 ▶), 541 Friedel pairs
Flack parameter: −0.08 (7)

### 

Data collection: *CrysAlis PRO* (Oxford Diffraction, 2003 ▶); cell refinement: *CrysAlis PRO*; data reduction: *CrysAlis RED* (Oxford Diffraction, 2003 ▶); program(s) used to solve structure: *SHELXS97* (Sheldrick, 2008 ▶); program(s) used to refine structure: *SHELXL97* (Sheldrick, 2008 ▶); molecular graphics: *SHELXTL* (Sheldrick, 2008 ▶) and *Mercury* (Macrae *et al.*, 2008 ▶); software used to prepare material for publication: *publCIF* (Westrip, 2010 ▶).

## Supplementary Material

Crystal structure: contains datablock(s) I, global. DOI: 10.1107/S1600536811054626/su2351sup1.cif

Structure factors: contains datablock(s) I. DOI: 10.1107/S1600536811054626/su2351Isup2.hkl

Supplementary material file. DOI: 10.1107/S1600536811054626/su2351Isup3.cml

Additional supplementary materials:  crystallographic information; 3D view; checkCIF report

## Figures and Tables

**Table 1:** Hydrogen-bond geometry (Å, °)

*D*—H⋯*A*	*D*—H	H⋯*A*	*D*⋯*A*	*D*—H⋯*A*
N3—H3⋯O6^i^	0.87 (1)	2.07 (1)	2.923 (2)	167 (2)
N1—H1⋯O2^ii^	0.86 (1)	2.05 (1)	2.881 (2)	165 (2)

Symmetry codes: (i) 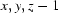; (ii) 
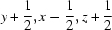
.

**Fig. 1.**
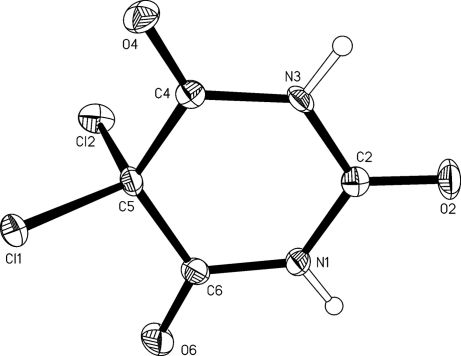
The molecular structure of the title compound, (Ic), with atom numbering. Displacement ellipsoids are drawn at the 50% probability level, with hydrogen atoms shown as spheres of arbitrary size.

**Fig. 2.**
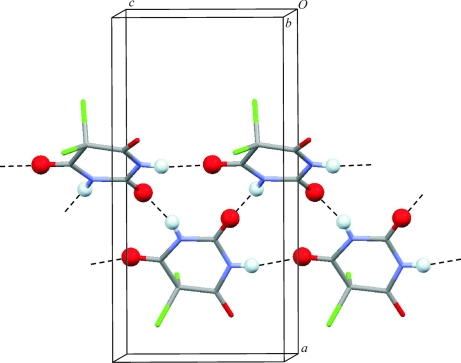
A view along the b axis of the H-bonded C-3 tape structure of (Ic). H, O and N atoms directly involved in N—H···O interactions (dashed lines) are drawn as balls.

**Fig. 3.**
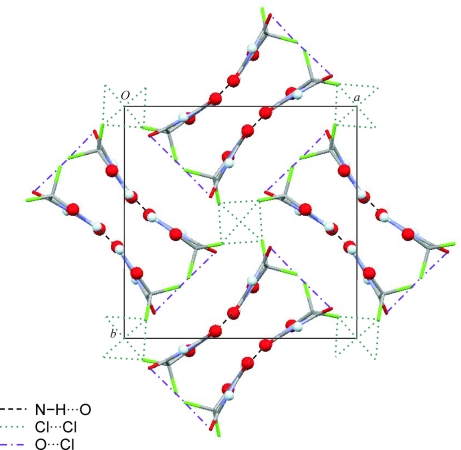
A portion of the crystal structure of (Ic), view along [001] (translation direction of the C-3 tapes) with four pairs of tapes. The N—H···O bonds and short intermolecular Cl···Cl and Cl···O contacts are indicated.

**Fig. 4.**
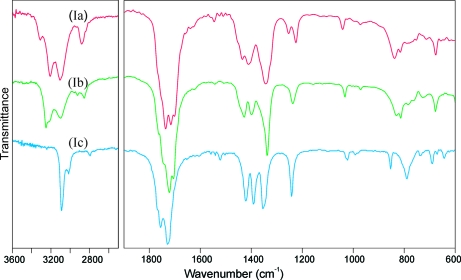
FT—IR spectra of the polymorphs (Ia), (Ib) and (Ic) of 5,5-dichlorobarbituric acid.

## supplementary crystallographic information

### Comment

The polymorphic nature of 5,5-dichlorobarbituric acid (I) is already mentioned in Groth's compendium on the chemical crystallography of organic compounds, published more than a hundred years ago (Groth, 1910). As part of our wider investigation of solid state forms of barbiturates, we have recently determined the crystal structures of a monoclinic (Ia) and an orthorhombic (Ib) form (Gelbrich *et al.*, 2011), and herein we report on the tetragonal polymorph (Ic) of the title compound. The equilibrium melting points of (Ia), (Ib) and (Ic), determined by hot-stage microscopy, are 477, 490 and 478 K, respectively. All three modifications were obtained in sublimation experiments; (Ia) as plates, (Ib) as prisms and (Ic) as long needles.

The molecular structure of (Ic) is illustrated in Fig. 1. The crystal structure of (Ic) consists of N—H···O═C bonded tapes (see Fig. 2) that belong to the C-3 type in the classification scheme proposed (Gelbrich *et al.*, 2011) for the H-bonded structures of 5,5-substituted derivatives of barbituratic acid*.* By contrast, all of the other five known crystal structures of 5,5-dihalogen analogues form either an N—H···O═C bonded layer (L) or a framework (F) structure (DesMarteau *et al.*, 1994; Gelbrich *et al.*, 2011). In particular, the monoclinic polymorph (Ia) and the orthorhombic form (Ib) display the layer types L-6 and L-5, respectively (Gelbrich *et al.*, 2011). C-3 tapes have been reported previously for solid forms of γ-methylamobarbital (Gartland & Craven, 1971), butobarbital (Gelbrich *et al.*, 2007), quinal barbitone (Nichol & Clegg, 2007) and alphenal (Zencirci *et al.*, 2009).

In the crystal structure of (Ic), a single tape consists of two parallel strands. Neighbouring molecules forming a single strand are N—H···O═C bonded to one another *via* their C6 carbonyl groups. Two strands of a tape are linked together by a second set of N—H···O═C interactions in which the C2 carbonyl group is involved. These interactions result in two independent *R*^3^_3_(12) rings (Etter *et al.*, 1990; Bernstein *et al.*, 1995). The molecules of a single strand are related to one another by a translation along [001]. Additionally, the tape possesses a glide mirror plane that is oriented perpendicular to its mean plane. The C4 carbonyl group is not involved in hydrogen bonding.

The cross section of the H-bonded tape structure is somewhat bent, so that the mean planes of the two strands from an angle of 23.7 (2)°. Two neighbouring H-bonded tapes, which are related to one another by a twofold rotation, form an assembly exhibiting short intermolecular Cl2···O4(–*y*+1/2, –*x*+1/2, *z*+1/2) distances of 3.201 (1) Å. As illustrated in Fig. 3, four such two-tape assemblies are situated around the fourfold roto-inversion axis in such a way that the Cl1 sites of four neighbouring molecules are the vertices of an almost ideal tetrahedron, whose edges are the intermolecular contacts Cl1···Cl1(–*y*+1, *x*, –*z*+1) and Cl1···Cl1(–*x*+1, –*y*+1, *z*) of 3.3873 (8) Å and 3.4462 (9) Å, respectively.

The three polymorphs of (I) can be readily distinguished from each other by their FT—IR spectra, which are depicted in Fig. 4. The calculated densities (Mg m^-3^) at -100 K for the three polymorphs (Ia), (Ib) and (Ic) are 1.984, 1.842 and 1.963, respectively. Therefore, the order of decreasing densities is (Ia) > (Ic) >> (Ib). The density of the tetragonal form (Ic) is 1% lower than that of the monoclinic form (Ia) and 6% higher than that of the orthorhombic polymorph (Ib), which is also the form of (I) with the most complex H-bonded structure.

### Experimental

Needle-shaped crystals of (Ic) were obtained in a sublimation experiment carried out at 473 K. On heating, (Ic) undergoes a transformation into (Ib). However, the melting of (Ic) can be observed in a thermomicroscopic experiment if the crystals are placed on a hot stage that is preheated to just below the melting temperature of (Ic).

The FT—IR spectrum of (Ic) (see Fig. 4) shows a strong and sharp N—H vibration at 3258 cm^-1^ and a weak one at 3152 cm^-1^. In the C═O region the spectrum exhibits a weaker band at 1756 cm^-1^ with a shoulder at about 1769 cm^-1^ and a stronger band at 1729 cm^-1^. These characteristics are consistent with the G5b-type spectrum in the IR classification schmeme for barabiturates (Zencirci *et al.*, 2009). This type indicates the presence of the H-bonded tape connectivity C-3. Previous G5b examples include form I of alphenal and the metastable polymorph VIII of phenobarbital (Zencirci *et al.*, 2009).

### Refinement

The NH H-atoms were located in a difference Fourier map. They were refined with a distance restraint: N—H = 0.88 (1) Å, with U_iso_(H) = 1.2U_eq_(N).

### Crystal data

**Table:**

C_4_H_2_Cl_2_N_2_O_3_	*D*_x_ = 1.963 Mg m^−^^3^
*M**_r_* = 196.98	Melting point: 478 K
Tetragonal, *P*42_1_*c*	Mo *K*α radiation, λ = 0.71073 Å
Hall symbol: P -4 2n	Cell parameters from 5173 reflections
*a* = 13.8883 (3) Å	θ = 2.9–29.3°
*c* = 6.9126 (2) Å	µ = 0.92 mm^−^^1^
*V* = 1333.34 (6) Å^3^	*T* = 173 K
*Z* = 8	Needle, colourless
*F*(000) = 784	0.20 × 0.05 × 0.05 mm

### Data collection

**Table:**

Oxford Diffraction Xcalibur Ruby Gemini ultra diffractometer	1310 independent reflections
Radiation source: Enhance Ultra (Mo) X-ray Source	1242 reflections with *I* > 2σ(*I*)
mirror	*R*_int_ = 0.041
ω scans	θ_max_ = 26.0°, θ_min_ = 2.9°
Absorption correction: multi-scan (*CrysAlis RED*; Oxford Diffraction, 2003)	*h* = −17→17
*T*_min_ = 0.837, *T*_max_ = 0.955	*k* = −16→17
11025 measured reflections	*l* = −7→8

### Refinement

**Table:**

Refinement on *F*^2^	Hydrogen site location: inferred from neighbouring sites
Least-squares matrix: full	All H-atom parameters refined
*R*[*F*^2^ > 2σ(*F*^2^)] = 0.020	*w* = 1/[σ^2^(*F*_o_^2^) + (0.0266*P*)^2^ + 0.2183*P*] where *P* = (*F*_o_^2^ + 2*F*_c_^2^)/3
*wR*(*F*^2^) = 0.050	(Δ/σ)_max_ < 0.001
*S* = 1.07	Δρ_max_ = 0.20 e Å^−^^3^
1310 reflections	Δρ_min_ = −0.16 e Å^−^^3^
107 parameters	Extinction correction: *SHELXL97* (Sheldrick, 2008), Fc^*^=kFc[1+0.001xFc^2^λ^3^/sin(2θ)]^-1/4^
2 restraints	Extinction coefficient: 0.0046 (8)
Primary atom site location: structure-invariant direct methods	Absolute structure: Flack (1983), 541 Friedel pairs
Secondary atom site location: difference Fourier map	Flack parameter: −0.08 (7)

### Special details

**Table:**

Geometry. All e.s.d.'s (except the e.s.d. in the dihedral angle between two l.s. planes) are estimated using the full covariance matrix. The cell e.s.d.'s are taken into account individually in the estimation of e.s.d.'s in distances, angles and torsion angles; correlations between e.s.d.'s in cell parameters are only used when they are defined by crystal symmetry. An approximate (isotropic) treatment of cell e.s.d.'s is used for estimating e.s.d.'s involving l.s. planes.
Refinement. Refinement of *F*^2^ against ALL reflections. The weighted *R*-factor *wR* and goodness of fit *S* are based on *F*^2^, conventional *R*-factors *R* are based on *F*, with *F* set to zero for negative *F*^2^. The threshold expression of *F*^2^ > σ(*F*^2^) is used only for calculating *R*-factors(gt) *etc*. and is not relevant to the choice of reflections for refinement. *R*-factors based on *F*^2^ are statistically about twice as large as those based on *F*, and *R*- factors based on ALL data will be even larger.

### Fractional atomic coordinates and isotropic or equivalent isotropic displacement parameters (Å^2^)

**Table:**

	*x*	*y*	*z*	*U*_iso_*/*U*_eq_	
Cl1	0.40679 (3)	0.41812 (3)	0.32982 (7)	0.01953 (13)	
Cl2	0.26242 (3)	0.27671 (3)	0.22840 (7)	0.01938 (13)	
O2	0.53042 (10)	0.10721 (9)	−0.1061 (2)	0.0225 (3)	
O4	0.37178 (9)	0.39086 (9)	−0.0966 (2)	0.0178 (3)	
O6	0.44620 (11)	0.22096 (11)	0.4808 (2)	0.0260 (4)	
N1	0.48923 (11)	0.16639 (11)	0.1869 (2)	0.0158 (3)	
N3	0.44480 (11)	0.24555 (11)	−0.0993 (2)	0.0127 (3)	
C2	0.49068 (12)	0.16895 (13)	−0.0125 (3)	0.0134 (4)	
C4	0.40209 (13)	0.32207 (12)	−0.0102 (3)	0.0120 (4)	
C5	0.38637 (12)	0.30971 (12)	0.2083 (3)	0.0131 (4)	
C6	0.44390 (12)	0.22935 (13)	0.3078 (3)	0.0148 (4)	
H1	0.5202 (13)	0.1184 (10)	0.233 (3)	0.018*	
H3	0.4508 (14)	0.2465 (15)	−0.2243 (14)	0.018*	

### Atomic displacement parameters (Å^2^)

**Table:**

	*U*^11^	*U*^22^	*U*^33^	*U*^12^	*U*^13^	*U*^23^
Cl1	0.0247 (2)	0.0140 (2)	0.0199 (2)	0.0036 (2)	−0.0065 (2)	−0.00722 (19)
Cl2	0.0156 (2)	0.0263 (2)	0.0162 (2)	−0.00433 (19)	0.00163 (18)	0.00387 (19)
O2	0.0286 (8)	0.0197 (7)	0.0193 (7)	0.0098 (6)	0.0067 (6)	−0.0037 (6)
O4	0.0212 (7)	0.0149 (7)	0.0172 (7)	0.0031 (5)	0.0002 (6)	0.0056 (6)
O6	0.0414 (9)	0.0266 (8)	0.0100 (7)	0.0112 (7)	−0.0030 (6)	0.0013 (6)
N1	0.0221 (8)	0.0124 (7)	0.0128 (8)	0.0072 (6)	−0.0019 (7)	0.0002 (7)
N3	0.0165 (8)	0.0151 (8)	0.0065 (7)	0.0004 (6)	0.0010 (6)	−0.0011 (7)
C2	0.0122 (9)	0.0134 (9)	0.0148 (10)	−0.0017 (7)	0.0013 (8)	0.0001 (8)
C4	0.0091 (9)	0.0132 (9)	0.0136 (9)	−0.0027 (7)	−0.0006 (8)	−0.0006 (7)
C5	0.0133 (8)	0.0130 (8)	0.0129 (9)	0.0006 (7)	0.0007 (7)	−0.0046 (7)
C6	0.0173 (9)	0.0140 (9)	0.0130 (10)	−0.0002 (7)	−0.0021 (7)	−0.0006 (8)

### Geometric parameters (Å, °)

**Table:**

Cl1—C5	1.7471 (18)	N1—H1	0.856 (9)
Cl2—C5	1.7868 (18)	N3—C4	1.364 (2)
O2—C2	1.208 (2)	N3—C2	1.378 (2)
O4—C4	1.203 (2)	N3—H3	0.868 (9)
O6—C6	1.202 (2)	C4—C5	1.536 (3)
N1—C6	1.363 (2)	C5—C6	1.535 (2)
N1—C2	1.379 (2)		
			
C6—N1—C2	127.07 (17)	N3—C4—C5	114.75 (16)
C6—N1—H1	120.1 (14)	C6—C5—C4	116.60 (15)
C2—N1—H1	112.9 (14)	C6—C5—Cl1	109.07 (12)
C4—N3—C2	127.31 (17)	C4—C5—Cl1	110.70 (13)
C4—N3—H3	118.6 (14)	C6—C5—Cl2	106.27 (12)
C2—N3—H3	113.6 (14)	C4—C5—Cl2	104.00 (12)
O2—C2—N3	121.74 (17)	Cl1—C5—Cl2	109.88 (10)
O2—C2—N1	121.60 (18)	O6—C6—N1	122.37 (18)
N3—C2—N1	116.65 (16)	O6—C6—C5	122.00 (17)
O4—C4—N3	123.15 (18)	N1—C6—C5	115.60 (16)
O4—C4—C5	121.84 (17)		

### Hydrogen-bond geometry (Å, °)

**Table:**

*D*—H···*A*	*D*—H	H···*A*	*D*···*A*	*D*—H···*A*
N3—H3···O6^i^	0.87 (1)	2.07 (1)	2.923 (2)	167.(2)
N1—H1···O2^ii^	0.86 (1)	2.05 (1)	2.881 (2)	165 (2)

Symmetry codes: (i) *x*, *y*, *z*−1; (ii) *y*+1/2, *x*−1/2, *z*+1/2.
